# Determinants of exclusive breastfeeding in infants less than six months of age in Hawassa, an urban setting, Ethiopia

**DOI:** 10.1186/s13006-017-0137-6

**Published:** 2017-11-02

**Authors:** Bethlihem Adugna, Henok Tadele, Fekadu Reta, Yifru Berhan

**Affiliations:** 1 Addis Ababa city administration Health Department, Kolfe Health Center, Addis Ababa, Ethiopia; 2 Department of Pediatrics and Child Health, College of Medicine and Health Sciences, Hawassa University, Hawassa, Ethiopia; 3 School of Nutrition, College of Agriculture, Hawassa University, Hawassa, Ethiopia; 4 Department of Obstetrics and Gynecology, College of Health Sciences, Addis Ababa University, Addis Ababa, Ethiopia

**Keywords:** Less than six months of age, Determinants, Ethiopia, Exclusive breastfeeding

## Abstract

**Background:**

The World Health Organization (WHO) recommends exclusive breastfeeding (EBF) for the first six months of life. However, the proportion of EBF in Ethiopia is 58%. The EBF practice and factors affecting it have not been studied in Hawassa, Southern Ethiopia. The aim of this study was to assess the prevalence and determinants of EBF practice among infants less than six months age in Hawassa city, Ethiopia.

**Methods:**

A total of 529 mothers with infants aged 0–6 months were involved in this study between November 2015 and January 2016. Trained interviewers collected data from the mothers of the infants. Exclusive breastfeeding was assessed based on infant feeding practice in the prior 24 h. Multivariable logistic regression analysis was conducted.

**Results:**

Infants aged 0–5.9 months were studied with comparable gender composition (51.4% females). The exclusive breastfeeding prevalence was 60.9% (95% CI 56.6, 65.1). Mothers with infants aged 0–1.9 months and 2–3.9 months practiced EBF more likely than mothers with infants aged 4–6 months (Adjusted odds ratio [AOR] 3.59; 95% CI 2.07, 6.2) and (AOR 2.08; 95% CI 1.23, 3.5), respectively. Married mothers practiced EBF more likely than singles (AOR 2.04; 95% CI 1.03, 4.06). Housewives practiced EBF more likely than employed mothers (AOR 2.57; 95% CI 1.34, 4.9). Mothers who had a vaginal birth were more likely to practice EBF than mothers who gave birth via Cesarean section (AOR 2.8; 95% CI 1.7, 4.6). Mothers who gave birth at a healthcare facility were more likely to practice EBF than mothers who gave birth at home (AOR 8.8; 95% CI 5.04, 15.4). Mothers without a breast complication practiced exclusive breastfeeding more than mothers with breast complications (AOR 2.05; 95% CI 1.5, 4.1).

**Conclusions:**

This study showed a low prevalence of exclusive breastfeeding. Younger infants, babies born to married women, who are housewives, having a vaginal birth in a health facility, and whose mother’s breasts were healthy, were predictors for EBF. The promotion of an institutional delivery, optimal breastfeeding practices, and designing strategies to better support employed mothers are recommended.

## Background

Breast milk is a safe and nutritive diet for the healthy growth and development of infants. The World Health Organization (WHO) recommends exclusive breastfeeding (EBF) for the first six months and continued breastfeeding for two years with the introduction of a complementary diet at six months of age [1]. The prevalence of EBF is below the international recommendation in many countries and worldwide, where only 36% of children less than six months of age are exclusively breastfed [2, 3].

Exclusive breastfeeding offers short term and long term health benefits both to the mother and the infant [4, 5]. It also provides economic benefits by reducing both the direct and indirect costs related to healthcare and infant feeding [6]. Non–exclusive breastfeeding is associated with poor academic achievement, and increased morbidity and mortality due to illnesses [7]. According to the recent Ethiopian Demographic and Health Survey (EDHS) report: plain water, non-milk liquids or other milk and complementary foods were given to 17%, 5% and 11% of infants aged 0–5 months in addition to breast milk, respectively. Breastfeeding used to be universal in Ethiopia but it has now dropped to 76% at 23 months of age. Only 58% of infants less than six months of age are exclusively breastfed. The percentage of exclusive breastfeeding decreased sharply with age, from 74% of infants at 0–1 month to 36% of infants at 4–5 months [8].

Several factors can affect the pattern of EBF practice; marital status, wealth index, and child’s age were determinant factors for EBF practice, according to the analysis done on the 2005 EDHS raw data [9]. Maternal unemployment and an infant aged less than two months determined EBF practice in a study conducted in South East Ethiopia [10]. A Ghanaian study has revealed higher practices of EBF among mothers who delivered at government health facility compared to mothers who delivered at home or at a private health facility [11].

The government of Ethiopia has initiated several interventions to optimize breastfeeding practices. Health information dissemination using different means, the preparation of training manuals and guidelines are among the intervention strategies. The health extension program, which functions at community level, also operates on infant feeding and nutrition program [12]. Non-governmental organizations are also addressing the issue of optimal breastfeeding in different regions of the country through advocacy, community mobilization, and mass communication [13]. The prevalence of EBF didn’t show a marked improvement despite the interventions. The level and determinant factors for exclusive breastfeeding practices have not been widely studied in southern Ethiopia, and none so far in Hawassa. Hence, the aim of the study was to identify the level of EBF practice and its determinants in Hawassa, southern Ethiopia. The study findings will assist program implementers in designing strategies for the promotion of exclusive breastfeeding in both the study and similar settings.

## Methods

### Study area

The study was conducted in Hawassa Tabor sub-city. Hawassa, the capital of Southern Nations and Nationalities Peoples’ Regional State, is located 275 Km south of Ethiopia’s capital city Addis Ababa. Hawassa has seven urban sub-cities and one rural sub-city. Tabor sub-city is the second largest sub-city only second to Tulla, a predominantly rural sub-city. Tabor sub-city has a population size of 72,115, with 2159 infants aged 0–6 months. Hawassa Tabor sub-city has five urban and suburban kebeles (the lowest administration unit in Ethiopia). It has one hospital, twenty clinics, one diagnostic laboratory, seven pharmacies, six medium and four higher level clinics.

### Study design

A community based cross-sectional study was conducted from November 2015 to January 2016 at Hawassa Tabor sub-city, Southern Ethiopia.

### Source and study population

All mothers with infants less than six months of age living in Hawassa Tabor sub-city for over six months were the source population.

### Inclusion and exclusion criteria

All mothers with infants less than six months of age were included in the study. Non-biologic, seriously ill mothers, non-consenting mothers and mothers unable to communicate due to hearing loss were excluded from the study.

### Sample size and sampling procedure

The sample size was calculated by using single population proportion formula by considering EBF prevalence of 49% [14]. The level of confidence was fixed as 95% with 4% margin of error and 15% nonresponse rate. Hence, the final calculated sample was 541 mothers with infants less than six months of age. All five kebeles in Hawassa Tabor sub-city were involved and the sample was allocated based on population proportion to size. A list of mothers with infants less than six months of age, was available at each kebele and used to identify the study participants by simple random sampling. Pretesting was done on 5% of the total respondents at a non-participating sub-city and kebele of Hawassa city, and adjustments were made to the interview tool accordingly. If the eligible mother was absent during the first visit, a second home visit was done, and if the mother was absent at the second visit, she was considered as non-respondent.

### Data collection

For data collection, pre-tested open-ended and close-ended questionnaires were used. Trained data collectors were deployed. Two days training on the data collection tool and procedure was given to eight data collectors and supervisors. Bachelor of Science (BSc) degree graduates in Nursing and Health Officer training were used for data collection and supervision. Completeness was assessed daily by the supervisors.

### Operational definitions

#### Exclusive breastfeeding

Mother breastfed and no other liquids or solids were given, to the child aged less than six months in the 24-h prior to the survey, with the exception of oral rehydration solution, supplements or medicines.

#### Predominant breastfeeding

The infant’s predominant source of nourishment has been breast milk, including milk expressed or feeding from a wet nurse as the predominant source of nourishment. However, the infant may also have received other liquids (water, water-based drinks, fruit juice), ritual fluids and ORS, drops or syrups (vitamins, minerals and medicines).

#### Partial breastfeeding

Giving a baby some breastfeeds, and some artificial feeds, either milk or cereal, or other food.

#### Seriously ill

Patients who are unconscious and unable to give the required information for this study.

#### Prelacteal feeding

Giving infants any drinks except medication or immunization and foods before the initiation of breast milk.

### Data analysis

The questionnaire was checked manually for completeness. Data were entered and analyzed using SPSS windows version 20. Descriptive statistics was computed to determine prevalence of EBF. Logistic regression analysis was carried at two levels to identify factors associated with EBF. First binary logistic regression analysis was carried out and variables with *p*-value <0.05 were included in the final multivariable logistic regression analysis. Strength of association was measured using odds ratio and 95% confidence intervals. The *p*-value <0.05 was set for statistical significance.

## Results

### Sociodemographic characteristics

In this study, a total of 529 mother-infant pairs participated, a response rate of 97.8%. The mean maternal age was 25.4 ± 4.24 years. Mothers aged 20–29 and 15–19 years accounted for 406 (76.7%) and 29 (5.5%) of the study subjects, respectively. Infant age ranged from 0 to 5.9 months with mean age of 3.05 ± 1.56 months and with comparable gender composition, 51.4% females. Married mothers constituted 475 (89.8%) of the study subjects. Maternal education status was found to be 51 (9.6%) illiterate, 187 (35.3%) completed primary education, 120 (22.7%) completed secondary education and 171 (32.3%) completed college and above. More than half of the mothers were multipara 297 (56.1%), primigravida and grand multipara accounted for 209 (39.5%) and 23 (4.3%), respectively. The mean family size of the study participants was 4.49 ± 1.76. The majority of the mothers were housewives, 303 (57.3%). More than half, 379 (71.6%) of the household monthly income was less than 1000ETB, (Table 1).

**Table 1:** Sociodemographic characteristics of respondents in Hawassa Tabor sub-city, 2016

Characteristics	Frequency (*n*)	Percentage (%)
Infant gender (*n* = 529)		
Male	257	48.6
Female	272	51.4
Infant age (*n* = 529)		
0–1.9	193	36.5
2–3.9	203	38.4
4–5.6	133	25.1
Maternal age (*n* = 529)		
15–19	29	5.5
20–29	406	76.7
30+	94	17.8
Marital status of the mother (*n* = 529)		
Married	475	89.8
Single	54	10.2
Maternal educational status (*n* = 529)		
Illiterate	51	9.6
Primary education	187	35.3
Secondary education	120	22.7
College and above	171	32.3
Parity (*n* = 529)		
Primigravida	209	39.5
Multipara	297	56.1
Grandmultipara	23	4.3
Family size (*n* = 529)		
2–3	184	34.8
4–5	244	46.1
Above 5	101	19.1
Maternal occupation (*n* = 529)		
House wife	303	57.3
Employee	226	42.7
Monthly income (*n* = 529)		
< = 1000 ETB	150	28.4
> 1000 ETB	379	71.6

### Obstetric and maternal health related characteristics

Health facility and home deliveries occurred in 414 (78.6%) and 113 (21.4%) of mothers, respectively. A vaginal birth occurred in 384 (72.6%) of the mothers. Three hundred thirty two (62.8%) mothers had antenatal care. Breast complications were reported by 111 (21%) of the mothers. Painful breasts were the most common reported complication, 56 (50.4%). From the mothers with breast complication, 18 (16.2%) reported that the problems had interfered with breastfeeding (Table 2).

**Table 2:** Obstetric and maternal health related characteristics of respondents in Hawassa Tabor sub-city, 2016

Characteristics	Frequency (*n*)	Percentage (%)
Place of delivery (*n* = 529)		
Health facility	416	78.6
Home	113	21.4
Type of delivery (529)		
Normal vaginal	384	72.6
Cesarean section	145	27.4
Antenatal care (*n* = 529)		
Yes	332	62.8
No	197	37.2
Mother sick in last two weeks (*n* = 529)		
Yes	74	14
No	455	86
Illnesses (*n* = 74)		
Back pain	12	16.2
Headache	6	8.1
Abdominal cramp	42	56.8
Others	14	18.9
Breast complication? (*n* = 529)		
Yes	111	21.0
No	418	79.0
Type of breast complication? (*n* = 111)		
Breast pain	56	50.6
Others	55	50.4
Breast problems prevent breastfeeding? (*n* = 111)		
Yes	18	16.2
No	93	83.7
How did it prevent? (*n* = 18)		
Interfered with EBF via early introduction of other solid/semisolid food	8	44.4
Reduced milk production	10	55.5

### Infant feeding practices based on 24 h-recall

Twenty four hours prior to the interview, other liquids were given to 35.1% of infants and formula milk, cow’s milk, and water accounted for 29.5%, 28.5% and 16.1%, respectively. Semisolids and solids were given to 7% of the infants. Breast milk only was given to 322 (60.9%) of the infants. The main reasons for giving other liquids and semisolid or solid foods were that the child feels hungry, breast milk alone was not enough, and advice from close relatives and others (Table 3).

**Table 3:** Infant feeding practices based on a 24 h-recall among respondents in Hawassa Tabor sub-city, 2016

Variables	Frequency (*n)*	Percentage (%)
Did you give liquids or fluids? (*n* = 529)		
Yes	186	35.1
No	343	64.8
Liquids or fluids given? (*n* = 186)		
Glucose	3	1.6
Boiled water	30	16.1
Formula	55	29.6
Cow’s milk	53	28.5
Medication	1	0.5
Others	44	23.6
Reason for giving liquids/fluids (*n* = 186)		
Baby felt hungry	71	38.2
Mother producing small milk	26	13.9
Advised by relatives	26	13.9
Advised by health professional	5	2.7
Advised by traditional birth attendant	5	2.7
To treat constipation	39	20.9
Others	14	7.5
Did you give semisolid/solid? (*n* = 529)		
Yes	37	7
No	492	93
Type of semisolid/solid foods? (*n* = 37)		
Egg	3	8.1
Fruit	6	16.2
Gruel	5	13.5
Mashed potato	8	21.6
Porridge	13	35.1
Others	2	5.4
Reason for giving semisolid/solid foods? (*n* = 37)		
Baby was hungry	23	62.1
Mother’s breast milk not enough	3	8.1
Advised by relatives	2	5.4
Advised by health professional	1	2.7
Advice by traditional birth attendant	3	8.1
To treat constipation	5	13.5
Breastfeeding category based on the 24 h recall (*n* = 529)		
Exclusively breastfeeding	322	60.9
Predominantly breastfeeding	65	12.3
Partial breastfeeding	142	26.8

### Prevalence of exclusive breastfeeding practice

Based on 24-h recall, the prevalence of EBF was 60.9% (95% CI 56.6, 65.1). The age specific prevalence of EBF was 42.9%, 39.1% and 18% at 1–1.9 months, 2–3.9 months, and 4–5.9 months, respectively. Predominant and partial breastfeeding was practiced in 12.3% and 26.8% of mothers, respectively.

### Knowledge and sources of information on breastfeeding

The majority of the mothers (80.7%) received counseling on breastfeeding. Breastfeeding counseling was obtained from health facilities, mass media, families or friends in 62.8%, 13.2% and 6.2% of the mothers, respectively. Breastfeeding counseling during antenatal, natal, and postnatal periods was obtained in 40.4%, 7% and 15.5%, respectively.

The majority of the mothers (96.4%) reported that breastfeeding protects the baby from illness. When asked about the timing of feeding, 95.1% replied that breastfeeding should be the baby’s first food and semi-solid/solid food should be introduced to the baby at six months of age. Concerning knowledge, 92.1% mothers reported that EBF could sustain a baby in a healthy condition for six months. Mothers (60.4%) reported that expressed breast milk is safe to feed to the baby when the mother is away.

### Factors associated with exclusive breastfeeding practices

Binary logistic regression analysis was done to select variables having association with EBF practice, and twelve variables were selected for final model analysis. Infant age, marital status, maternal occupation, place of birth, mode of delivery and presence of breast complication were significantly associated with EBF practice among infants less than six months of age in multivariable logistic regression analysis.

The practice of EBF among infants aged 0–1.9 months and 2–3.9 months was 3.59 times (95% CI 2.07, 6.2) and 2.08 times (95% CI 1.23, 3.5) more likely than those infants aged 4–6 months, respectively. Married mothers practiced EBF 2.04 times more likely than single mothers (95% CI 1.03, 4.06). Housewives practiced EBF 2.57 times more likely than employed mothers (95% CI 1.34, 4.9).

Mothers who gave birth at a health facility practiced exclusive breastfeeding 8.8 times more than (AOR 8.8; 95% CI 5.04, 15.4) mothers who gave birth at home. Mothers who gave birth naturally exclusively breastfed their infants 2.8 times more likely than (AOR 2.8; 9% CI 1.7, 4.6) mothers with Cesarean section. Concerning breast complications, mothers with no breast complication practiced EBF 2.5 times more likely than (AOR 2.5; 95% CI 1.5, 4.1) mothers with breast complications (Table 4).

**Table 4:** Determinants of EBF practice among mothers with infants less than 6 months of age in Hawassa Tabor Sub-city, 2016

Variables	EBF*n* (%)	NEBF *n* (%)	Crude odds ratio (95% CI)	Adjusted odds ratio (95% CI)
Infant gender (*n* = 529)				
Male	169 (52.5)	88 (42.5)	1.49 (1.05,2.1)*	1.41 (0.92,2.15)
Female	153 (47.5)	119 (57.5)	1	1
Infant age in months (*n* = 529)				
0–1.9	138 (42.9)	55 (26.6)	3.24 (2.04,5.15)**	3.59 (2.07,6.2)**
2–3.9	126 (39.1)	77 (37.2)	2.11 (1.35,3.3)*	2.08 (1.23,3.5)*
4–5.9	58 (18)	75 (36.2)	1	1
Marital status (*n* = 529)				
Married	298 (92.5)	177 (85.5)	2.1 (1.19,3.71)*	2.04 (1.03,4.06)*
Single	24 (7.5)	30 (14.5)	1	1
Maternal education status (*n* = 529)				
Illiterate	31 (9.6)	20 (9.7)	1.46 (0.7,2.7)	1.44 (0.57,3.62)
Primary education	124 (38.5)	63 (30.4)	1.85 (1.2, 2.8)*	1.50 (0.79,2.85)
Secondary education	79 (24.5)	41 (19.8)	1.81 (1.12,2.9)*	1.09 (0.55,2.16)
College and above	88 (27.3)	83 (40.1)	1	1
Maternal occupation (*n* = 529)				
Housewife	208 (64.6)	95 (45.9)	2.15 (1.5,3.07)**	2.57 (1.34,4.9)*
Employee	114 (35.4)	112 (54.1)	1	
Maternal income source (*n* = 357)				
Salary	71 (22)	65 (31.4)	1	1
Husband	19 (59.3)	100 (48.3)	1.74 (1.15,2.64)*	0.83 (0.39,1.77)
Self-business	60 (18.6)	42 (20.3)	1.3 (0.77,2.19)	1.31 (0.63,2.7)
Place of delivery (*n* = 529)				
Home	33 (10.2)	80 (38.6)	1	1
Health facility	289 (89.8)	127 (61.4)	5.5 (3.4,8.7)**	8.8 (5.04,15.4)**
Type of delivery (*n* = 529)				
Normal vaginal	249 (77.3)	135 (65.2)	1.81 (1.23,2.6)*	2.8 (1.7,4.6)**
Caesarian section	73 (22.7)	72 (34.8)	1	1
Breast complication (*n* = 490)				
Yes	45 (14)	66 (31.9)	1	1
No	289 (89.8)	90 (43.5)	2.88 (1.87,4.42)*	2.5 (1.5,4.1)**
BF is alone enough for six months				
Yes	307 (95.3)	12 (5.8)	3.00 (1.59,5.9)*	2.57 (1.34,4.9)*
No	15 (4.7)	180 (87)	1	1
Information on optimal breastfeeding (*n* = 529)				
Yes	278 (86.3)	149 (72)	2.45 (1.58,3.8)**	1.66 (0.85,3.25)
No	44 (13.7)	58 (28)	1	1
Antenatal care (*n* = 529)				
No	101 (31.4)	96 (46.4)	1	1
Yes	221 (68.6)	111 (53.6)	1.89 (1.31,2.7)*	0.92 (0.53,1.6)

*p < 0.05)s *p* < 0.001

*1* = reference; *EBF* = exclusive breastfeeding; *NEBF* = Nonexclusive breastfeeding; *CI* = Confidence interval

## Discussion

The prevalence of EBF in this study was 60.9%. This is higher than a study done in Bahirdar, (50.3%) [15], and lower than studies done in Goba district (71.3%) and Tigray region (70.2%) in Ethiopia [10, 16]. This result is far from Ethiopia’s fifth Health Sector Development program (HSDP IV) set target, of 70% [17]. Inclusion of urban and rural setting in the above studies and cross cultural differences could have attributed to the difference. The recall since birth method used to determine exclusive breastfeeding in the Bahirdar study might have underestimated the EBF prevalence.

In our study, the practice of exclusive breastfeeding was associated with infant age. The practice of EBF decreased significantly, close to six months of age, this is in agreement with studies conducted in Brazil, Nigeria and Ethiopia [9, 18–20]. The traditional postpartum family care given in the first few months when mothers remain at home, might encourage exclusive breastfeeding [9]. Complementary feeding might be introduced to infants with the assumption that breast milk alone will not satisfy the need of infant as they approach six months of age.

In the present study, housewives practiced EBF more than employed mothers and this is similar to studies done in Malaysia, Sudan and Ethiopia [21–23]. Early return of employed mothers to the office, lack of support from the office and short maternity leave (only three months paid leave in Ethiopian case) could also discourage employed mothers.

Concerning the place of delivery, mothers who delivered at health facilities practiced EBF more likely than mothers who delivered at home. This is similar to studies done in Ghana, Tanzania, and Nigeria [11, 24, 25]. A Canadian study revealed the opposite results and the negative effect of formula supplementation in their setting was pointed out as the reason [26]. Our finding reinforces the international recommendation of making health facilities ‘baby-friendly’ as information received from healthcare facilities is welcomed by parents and society.

In this study, more mothers who gave birth naturally practiced exclusive breastfeeding compared to mothers following a Caesarian section delivery. This finding is similar to studies done in Canada, Nepal, and Ethiopia [15, 26, 27]. The effects of Caesarian section on maternal and fetal stress response and disrupted lactogenesis especially in the first 12 weeks postpartum, are reported as a cause for an unsuccessful first breastfeeding attempt and inability to breastfeed upon leaving the healthcare facility [28]. Earlier discharge from the healthcare facility of mothers who had a vaginal birth and their reattachment with families could make the postpartum care smooth and increase chances of EBF.

In the current study, the presence of breastfeeding complications was associated with EBF practice. This finding is in agreement with a study done in Mauritius [29]. An Indian study has documented the absence of any association between breast problems and EBF, however it was done at single healthcare facility [30]. Our finding underscores the need for proper education on optimal breastfeeding techniques and practices to avoid breast problems and the subsequent impact on exclusive breastfeeding. Though our method of EBF assessment has no recall bias, it may overestimate the rate of exclusive breastfeeding [31, 32].

## Conclusion

This study showed a low prevalence of exclusive breastfeeding. Being a younger infant, born to a married woman, who was a housewife, a vaginal birth in a health facility, and healthy mother’s breast were determinant factors for EBF. The promotion of a health facility and vaginal birth, optimal breastfeeding practices, and strategies designed to better support employed mothers are recommended.

## Abbreviations

AOR: Adjusted odds ratio
CI: Confidence interval
COR: Crude odds ratio
EBF: Exclusive breastfeeding
EDHS: Ethiopian demographic health survey
ETB: Ethiopian birr
HSDP/HSTP: health sector development/transformation program/plan
NEBF: Non - exclusive breastfeeding
SNNPR: Southern Nations and Nationalities People’s Regional State
SPSS: Statistical package for social science
WHO: World Health Organization
